# Long-leg spica casts demonstrate superior outcomes in developmental dysplasia of the hip: a multi-center retrospective analysis of failure risks and protective factors

**DOI:** 10.3389/fped.2025.1563536

**Published:** 2025-04-01

**Authors:** Zibing Zheng, Weiguang Yu, Zhao Chen, Jinrun Lin, Jinluan Lin, Hui Chen

**Affiliations:** ^1^ Department of Pediatric Orthopedics, Fujian Children’s Hospital (Fujian Branch of Shanghai Children’s Medical Center), College of Clinical Medicine for Obstetrics & Gynecology and Pediatrics, Fujian Medical University, Fuzhou, China; ^2^ Department of Orthopedics, The First Affiliated Hospital, Sun Yat-sen University, Guangzhou, China; ^3^ Department of Orthopedics, The First Affiliated Hospital of Fujian Medical University, Fuzhou, China; ^4^ Department of Pediatric Orthopedics, Fujian Children’s Hospital (Fujian Branch of Shanghai Children’s Medical Center), College of Clinical Medicine for Obstetrics & Gynecology and Pediatrics, Fujian Medical University, Fujian Maternity and Child Health Hospital, Fuzhou, China

**Keywords:** cast, dysplasia, spica, survival, developmental dysplasia of the hip

## Abstract

**Objectives:**

While both short- and long-leg spica casts present viable options after closed reduction (CR) in developmental dysplasia of the hip (DDH), comprehensive comparative studies are needed to guide clinical practices. This multi-center retrospective study aimed to evaluate their effectiveness in treating DDH and identify predictors of treatment failure.

**Methods:**

This retrospective study analyzed 146 DDH patients (0–18 months) treated with closed reduction and spica casts (70 short-leg vs. 76 long-leg) at two tertiary centers (2005–2024). Incident-free survival (time from casting to failure: re-dislocation, imaging-confirmed reduction loss, or surgical conversion) was analyzed via Kaplan–Meier/Log-rank tests. A multivariable Cox model evaluated eight variables: cast type, age (>6 vs. ≤6 months), sex, laterality (bilateral/unilateral), IHDI grade (IV/III), birth presentation, delivery mode, and family history, allowing quantification of independent predictors associated with treatment failure risks.

**Results:**

The analysis of 146 DDH cases (70 short-leg vs. 76 long-leg casts) revealed significant outcome differences between cast types. Patients receiving long-leg spica casts demonstrated substantially higher 6-month incident-free survival rates (84% vs. 68%, Log-rank *p* < 0.05), with multivariable Cox regression confirming long-leg casting as an independent protective factor (HR = 0.45, 95% CI 0.32–0.64, *p* < 0.001). The analysis identified three significant risk predictors: older age (>6 months) increased failure risk by 89% (HR = 1.89, 95% CI 1.02–3.51), bilateral involvement elevated risk by 78% (HR = 1.78, 95% CI 1.25–2.54), and IHDI IV dysplasia doubled failure likelihood (HR = 2.15, 95% CI 1.45–3.18). Notably, cephalic presentation showed a protective trend (HR = 0.67, 95% CI 0.48–0.93), while delivery mode and family history did not reach statistical significance.

**Conclusions:**

Long-leg spica casting shows superior biomechanical stability in DDH management post-CR, particularly for high-risk patients. These findings support its preferential use in patients with bilateral involvement, advanced dysplasia, or older age (>6 months).

## Introduction

Developmental dysplasia of the hip (DDH) is a significant orthopedic condition in pediatrics, characterized by a spectrum from mild dysplasia to complete dislocation of the hip joint (1–3). Early intervention is crucial to prevent long-term disabilities (4, 5). When nonoperative methods such as harnesses fail, closed reduction (CR) is the standard treatment (6, 7). Post-CR immobilization is pivotal for maintaining anatomic hip positioning and fostering normal joint development (8, 9). While long-leg spica casts (extending below the knee) remain traditional for stabilization, their biomechanical advantages are counterbalanced by mobility and hygiene challenges (10, 11). Short-leg spica casts (ending above the knee) have gained traction as a patient-friendly alternative, yet conflicting evidence exists regarding their efficacy compared to long-leg casts (12). Prior studies, limited by small cohorts, report comparable short-term outcomes, but critical knowledge gaps persist regarding long-term stability, failure risks, and predictors such as age (>6 months), bilateral involvement, or severe dysplasia (IHDI grade IV).

This multi-center study evaluates 377 DDH patients (2005–2024) to compare post-CR outcomes between short- and long-leg spica casts, while identifying independent protective factors (e.g., cast type) and risk predictors (e.g., age, laterality, dysplasia severity). By addressing these gaps, we aim to refine evidence-based, patient-centered immobilization strategies for high-risk subgroups. This study hypothesizes that long-leg spica casts provide superior biomechanical stability compared to short-leg casts, resulting in higher incident-free survival and lower complication rates post-CR for DDH.

## Materials and methods

### Study design and participants

This multi-center retrospective cohort study analyzed data from Fujian Medical University's Affiliated Children's Hospital and the First Affiliated Hospital of Sun Yat-sen University (January 2005–December 2024), focusing on pediatric patients with DDH treated with CR followed by short-leg (above-knee) or long-leg (below-knee) spica casts. Conducted in compliance with ethical standards and institutional review board approval, this research involved an extensive review of electronic medical records. Inclusion criteria encompassed consecutive patients aged 0–18 months with diagnosis of DDH, treated with CR and spica cast immobilization, having complete medical records.

Radiographic severity was classified using the Tönnis system and International Hip Dysplasia Institute (IHDI) criteria, particularly for cases with an absent ossific nucleus. Patients classified as Tönnis grades I–II or IHDI grades I–II were excluded, as these mild-to-moderate dysplasias typically resolve with bracing alone and do not necessitate CR. Additional exclusions: age >18 months, prior hip surgery/DDH treatment, congenital anomalies affecting the hip, or incomplete medical or follow-up records.

### Data collection and variables

Demographics (age, sex), birth history (presentation, delivery mode), family DDH history, hip laterality (unilateral/bilateral), and cast type (short- vs. long-leg) were extracted. Two blinded pediatric orthopedic surgeons independently evaluated imaging studies, with discrepancies resolved by consensus. Eight clinically relevant variables were analyzed for failure risk: cast type, age (>6 vs. ≤6 months), sex, laterality, Tönnis grade (IV vs. III), birth presentation (breech/cephalic), delivery mode (cesarean/vaginal), and family DDH history.

### Outcome measures

The primary outcomes focused on incident-free survival and risk predictors. Incident-free survival was defined as the time interval from cast application to the first treatment failure event, including re-dislocation, imaging-confirmed reduction loss, or conversion to open surgery. This outcome was analyzed using Kaplan–Meier survival curves with Log-rank testing for comparative assessment. Risk predictors were evaluated through multivariable Cox proportional hazards regression models to quantify hazard ratios (HRs) for treatment failure while adjusting for covariates.

Secondary outcomes addressed clinical and patient-centered metrics. Acute reduction success was confirmed intraoperatively via arthrography to verify concentric reduction and postoperatively with computed tomography (CT) within 24 h to assess femoral head alignment and acetabular coverage (13, 14). Long-term stability at 12 months post-CR was evaluated using pelvic radiographs, applying thresholds such as an acetabular index ≤25° (normal: ≤20°–25° in children <2 years) (15–17) and Reimer's migration index <20% to confirm stable femoral head coverage (18–20). Complications included residual dysplasia (acetabular index >30°) (21) and avascular necrosis [AVN, classified using the Kalamchi-MacEwen system grades I–IV (22): Grade I (temporary growth disturbance), Grade II (lateral physeal damage), Grade III (central physeal collapse), and Grade IV (total femoral head involvement)]. Reoperations were documented for cases requiring secondary procedures due to treatment failure or complications. Patient-centric outcomes incorporated caregiver-reported Likert-scale assessments of mobility limitations, hygiene challenges, and comfort levels (18, 21, 22).

### Statistical analysis

Descriptive statistics summarized demographic and clinical variables. Group comparisons (short- vs. long-leg casts) employed chi-square tests for categorical variables and *t*-tests or Mann–Whitney *U* tests for continuous variables, selected based on normality (assessed via Shapiro–Wilk tests). Kaplan–Meier curves with Log-rank tests compared incident-free survival between cast types. Multivariable Cox regression (forward stepwise selection, *α* = 0.05) identified independent predictors, with results reported as HRs and 95% confidence intervals (CIs). Proportional hazards assumptions were verified via Schoenfeld residuals. Significance was set at *p* < 0.05. Analyses were performed using R v.4.4.2 (R Foundation).

## Results

**Figure 1:**
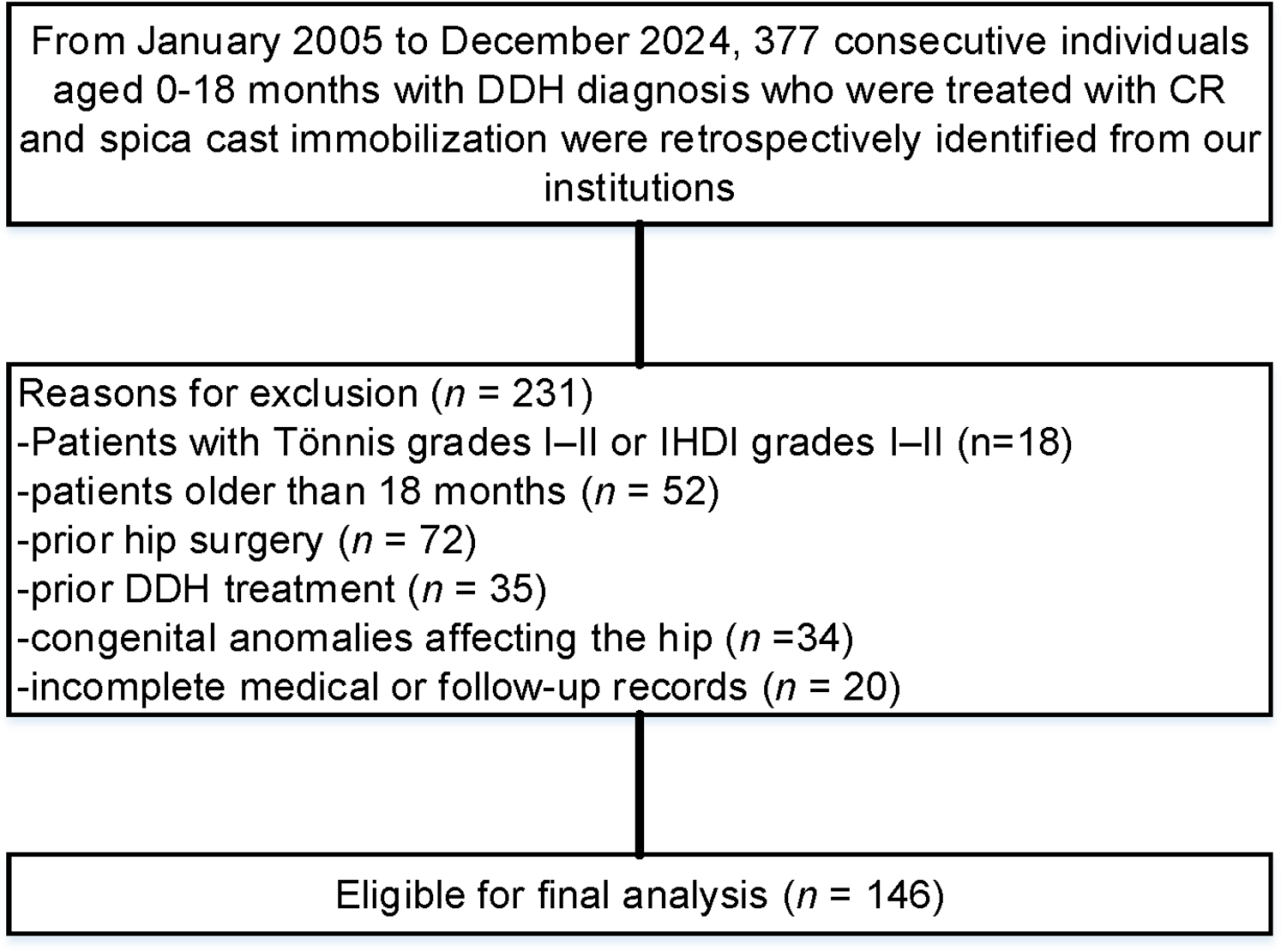
Flow diagram demonstrating the methods used to identify objects to evaluate the effectiveness of short- and long-leg spica casts in treating DDH.

The study analyzed 146 pediatric DDH patients treated with CR followed by spica casting, including 70 in the short-leg cast group and 76 in the long-leg cast group (Figure 1). Baseline characteristics were comparable between cohorts (Table 1), with no significant differences in age distribution (≤6 months: 31% short-leg vs. 26% long-leg, *p* = 0.497), sex (54% short-leg vs. 53% long-leg, *p* = 0.842), laterality (bilateral: 51% short-leg vs. 46% long-leg, *p* = 0.518), or IHDI grade IV severity (20% short-leg vs. 24% long-leg, *p* = 0.592). Birth presentation, delivery mode, and family history showed no significant imbalances (all *p* > 0.05).

**Table 1:** Patient characteristics at baseline between two cohorts.

Variable	Short-leg spica (*n* = 70)	Long-leg spica (*n* = 76)	*p*-value^a^
Age, months, no. (%)			
≤6 months	22 (31)	20 (26)	0.497
>6 months	48 (69)	56 (74)	
Sex, no. (%)			0.842
Female	38 (54)	40 (53)	
Male	32 (46)	36 (47)	
Site, no. (%)			0.518
Unilateral	34 (49)	41 (54)	
Bilateral	36 (51)	35 (46)	
Tonnis and IHDI classifications, no. (%)			0.592
Grade III	56 (80)	58 (76)	
Grade IV	14 (20)	18 (24)	
Presentation, no. (%)			0.460
Breech	40 (57)	48 (63)	
Cephalic	30 (43)	28 (37)	
Mode of delivery, no. (%)			0.502
Normal vaginal deliver	60 (86)	62 (82)	
Caesarean section	10 (14)	14 (18)	
Family history, no. (%)			0.358
Yes	9 (13)	14 (18)	
No	61(87)	62(82)	

IHDI, international hip dysplasia institute.

^a^ Analysed using Mann–Whitney *U* test.

**Figure 2:**
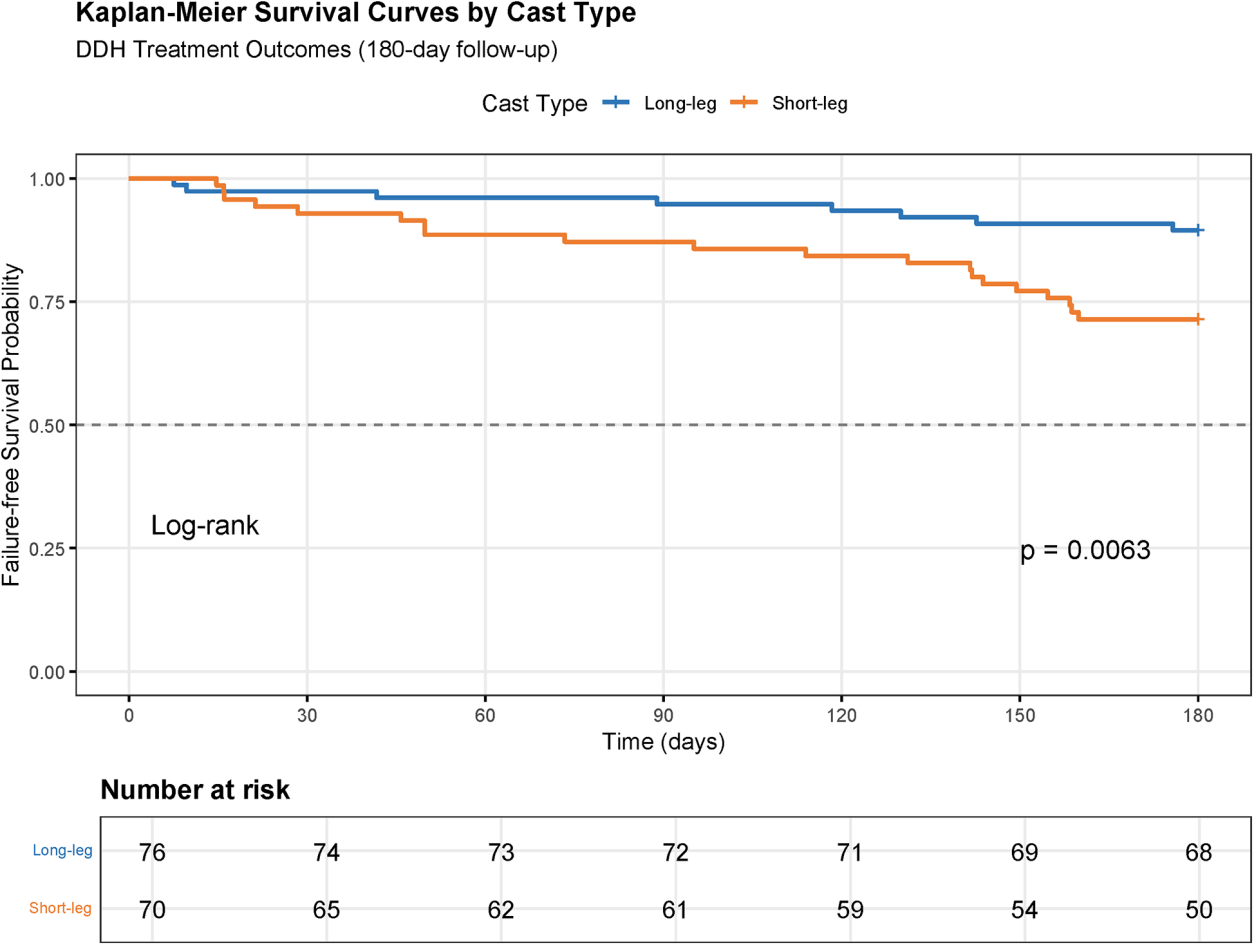
Survival analysis of long-leg cast versus short-leg cast in DDH.

Long-leg spica casts demonstrated superior clinical outcomes. Kaplan–Meier analysis revealed significantly higher 6-month incident-free survival rates in the long-leg group (84% vs. 68%, Log-rank *p* < 0.05) (Figure 2). Multivariable Cox regression identified long-leg casting as an independent protective factor against treatment failure (HR = 0.45, 95% CI 0.32–0.64, *p* < 0.001). Conversely, age >6 months (HR = 1.89, 95% CI 1.02–3.51, *p* = 0.001), bilateral involvement (HR = 1.78, 95% CI 1.25–2.54, *p* = 0.001), and IHDI grade IV dysplasia (HR = 2.15, 95% CI 1.45–3.18, *p* < 0.001) were significant risk predictors. Cephalic birth presentation showed a protective trend (HR = 0.67, 95% CI 0.48–0.93, *p* = 0.018), while delivery mode and family history lacked statistical significance (Table 2).

**Table 2:** Cox regression analysis to assess the correlation between cast type (short vs. long spica) and outcomes.

Covariate	HR	SE	95% CI Lower	95% CI Upper	*p-*value
Long-leg	0.45	0.42	0.32	0.64	<0.001
Age >6 months	1.89	1.56	1.02	3.51	0.001
Male	1.52	0.76	0.35	1.12	0.078
Bilateral	1.78	1.75	1.25	2.54	0.001
IHDI IV	2.15	2.12	1.45	3.18	<0.001
Cephalic	0.67	1.12	0.88	1.93	0.072
Caesarean section	1.35	1.54	0.95	1.92	0.092
Family history, Yes	1.28	1.05	0.89	1.84	1.186

HR, hazard ratio; SE, standard error; CI, confidence interval.

The long-leg spica group demonstrated superior acute success rates [89% (68/76)] compared to the short-leg spica group [71% (50/70); *p* = 0.006]. Furthermore, the incidence of residual deformity was significantly lower in the long-leg spica cohort [15% (12/76)] than in the short-leg spica group [34% (24/70); *p* = 0.01]. Notably, avascular necrosis (AVN) occurred less frequently in the long-leg spica group [9% (7/76)] compared to the short-leg spica cohort [23% (16/70); *p* = 0.024] (Table 3). These results underscore the clinical advantages of long-leg spica fixation in reducing complications and improving acute treatment success for the studied population.

**Table 3:** Comparison of clinical outcomes between two cohorts.

Variable	Short-leg spica (*n* = 70)	Long-leg spica (*n* = 76)	*p*-value^a^
Acute Success	71% (50/70)	89% (68/76)	0.006
Residual Deformity	34% (24/70)	15% (12/76)	0.01
AVN	23% (16/70)	9% (7/76)	0.024

AVN, avascular necrosis.

^a^ Analysed using Mann–Whitney *U* test.

## Discussion

The findings of this multicenter retrospective analysis provide compelling evidence for the biomechanical superiority of long-leg spica casts in maintaining hip stability following CR for DDH. Our results demonstrate that long-leg spica casting significantly outperforms short-leg casting in both acute success rates and long-term incident-free survival. The Kaplan–Meier analysis revealed a marked disparity in 6-month incident-free survival (84% vs. 68%, Log-rank *p* < 0.05), corroborated by multivariable Cox regression identifying long-leg casts as an independent protective factor (HR = 0.45, *p* < 0.001). This aligns with biomechanical principles positing that immobilization extending below the knee enhances femoral head containment by limiting rotational and translational forces across the hip joint, thereby reducing risks of reduction loss (23, 24). Early studies advocating short-leg casts emphasized patient comfort and mobility (25), yet our data challenge the assumption of equivalence in stabilization efficacy. The 19% absolute risk reduction in residual deformity (15% vs. 34%, *p* = 0.01) and 14% reduction in avascular necrosis (AVN, 9% vs. 23%, *p* = 0.024) further underscore the clinical relevance of extended immobilization.

Contrary to previous reports (26, 27) suggesting comparable AVN risks between cast types, our cohort revealed significantly lower AVN incidence in the long-leg group. This discrepancy may stem from differences in preoperative protocols, such as traction duration or reduction techniques, which were standardized in our study. Recent studies (12, 28) reported no association between cast length and AVN, their smaller sample size and heterogeneous cohorts may have obscured true differences. Our findings suggest that biomechanical stability conferred by long-leg casts indirectly mitigates ischemic risks by minimizing microtrauma from residual instability, a hypothesis supported by animal models demonstrating vascular compromise during repetitive joint loading (29, 30).

The argument for short-leg casts as a patient-friendly alternative must be weighed against their inferior clinical outcomes. Although researchers reported improved hygiene and mobility with short-leg casts in prior studies (14), these benefits did not translate to superior functional outcomes in our cohort. Instead, the high complication rate and need for surgical conversion in the short-leg group highlight the critical trade-off between convenience and efficacy. Notably, the protective trend associated with cephalic presentation (HR = 0.67, *p* = 0.072) and the elevated risks linked to bilateral involvement (HR = 1.78) and IHDI grade IV dysplasia (HR = 2.15) further emphasize the need for robust immobilization in high-risk subgroups.

Several limitations merit detailed discussion. First, the retrospective design of most included studies introduces selection bias and confounds causal inference. Second, heterogeneous patient cohorts, variable treatment protocols, and inconsistent outcome measures complicate cross-study comparisons. Finally, the predominance of small sample sizes and limited long-term follow-up restricts the generalizability of findings. Future prospective multicenter studies with standardized endpoints—including patient-reported outcomes and advanced imaging metrics—are essential to refine immobilization protocols and optimize individualized care.

## Conclusion

In light of the significantly higher 6-month incident-free survival (84% vs. 68%) and reduced complications observed with long-leg spica casts, we recommend against the use of short-leg casts for DDH patients aged 0–18 months requiring post-CR immobilization. While short-leg casts may offer logistical advantages, their association with higher residual deformity, AVN, and failure rates necessitates prioritizing biomechanical stability over transient comfort. Long-leg spica casting should be the standard of care, particularly for patients with bilateral involvement, severe dysplasia (IHDI IV), or age >6 months, where failure risks are amplified. Future studies should explore hybrid immobilization strategies that balance stability with patient-centered outcomes, ensuring adherence without compromising therapeutic efficacy.

## Data Availability

The original contributions presented in the study are included in the article/Supplementary Material, further inquiries can be directed to the corresponding authors.
